# Seed abortion caused by the combination of two duplicate genes in the progeny from the cross between *Oryza sativa* and *Oryza meridionalis*

**DOI:** 10.1270/jsbbs.23084

**Published:** 2024-04-02

**Authors:** Daiki Toyomoto, Yukika Shibata, Masato Uemura, Satoru Taura, Tadashi Sato, Robert Henry, Ryuji Ishikawa, Katsuyuki Ichitani

**Affiliations:** 1 United Graduate School of Agricultural Sciences, Kagoshima University, 1-21-24 Korimoto, Kagoshima, Kagoshima 890-0065, Japan; 2 Graduate school of Agriculture, Forestry and Fisheries, Kagoshima University, 1-21-24 Korimoto, Kagoshima, Kagoshima 890-0065, Japan; 3 Graduate school of Agriculture, Kagoshima University, 1-21-24 Korimoto, Kagoshima, Kagoshima 890-0065, Japan; 4 Institute of Gene Research, Kagoshima University, 1-21-24 Korimoto, Kagoshima, Kagoshima 890-0065, Japan; 5 Graduate School of Agricultural Science, Tohoku University, Sendai, Miyagi 980-8572, Japan; 6 Queensland Alliance for Agriculture and Food Innovation, University of Queensland, Brisbane, Queensland, 4072, Australia; 7 Faculty of Agriculture and Life Science, Hirosaki University, Hirosaki, Aomori 036-8561, Japan; 8 Faculty of Agriculture, Kagoshima University, 1-21-24 Korimoto, Kagoshima, Kagoshima 890-0065, Japan

**Keywords:** reproductive isolation, seed abortion, *O. meridionalis*, chromosomal duplication, recessive lethal genes, fine mapping

## Abstract

Seed development is an essential phenomenon for all sexual propagative plant species. The functional allele at *SEED DEVELOPMENT 1* (*SDV1*) or *SEED DEVELOPMENT 2* (*SDV2*) loci is essential for seed development for *Oryza sativa* and *Oryza meridionalis*. In the present study, we performed fine mapping of *SDV1*, narrowing down the area of interest to 333kb on chromosome 6. Haplotype analysis around the *SDV1* locus of *O. meridionalis* accessions indicated that they shared the DNA polymorphism, suggesting that they have a common abortive allele at the *SDV1* locus. Linkage analysis of the candidate *SDV2* gene showed that it was located on chromosome 4. The candidate *SDV2* was confirmed using a population in which both the *SDV1* and *SDV2* genes were segregating. The chromosomal region covering the *SDV1* gene was predicted to contain 30 protein-coding genes in *O. sativa*. Five of these genes have conserved DNA sequences in the chromosomal region of the *SDV2* gene on chromosome 4, and not on chromosome 6, of *O. meridionalis*. These results suggest that these five genes could be candidates for *SDV1*, and that their orthologous genes located on chromosome 4 of *O. meridionalis* could be candidates for *SDV2*.

## Introduction

**Fig. 1.**
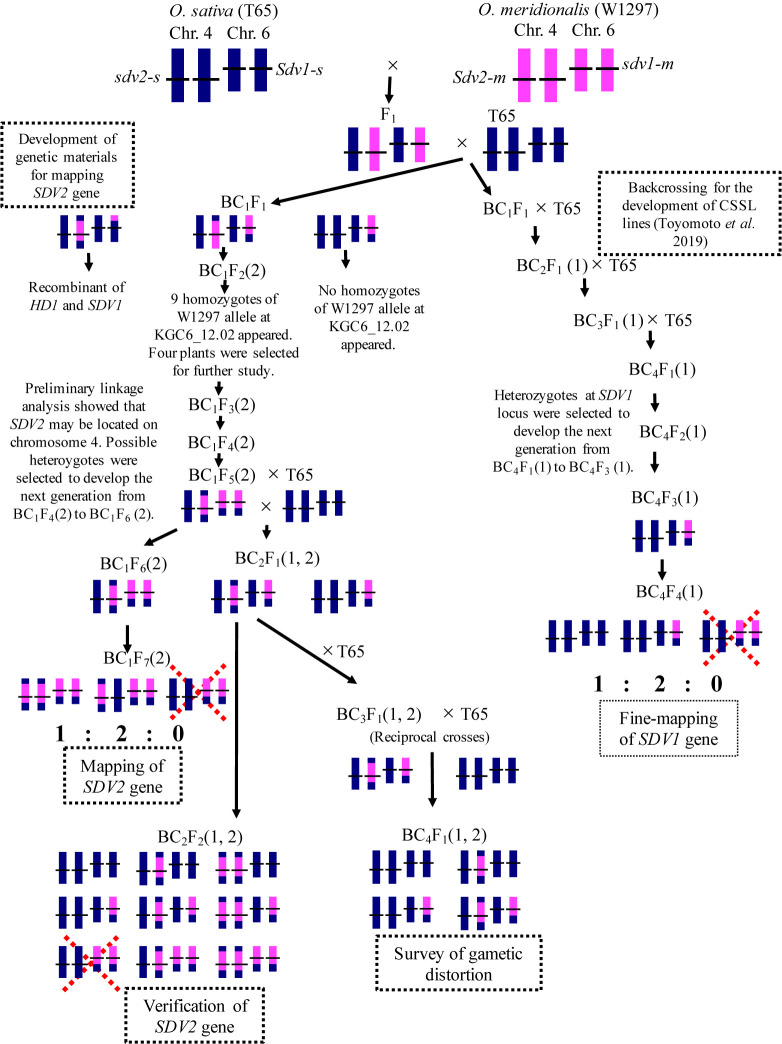
The genetic model of seed fertility controlled by S*DV1* and *SDV2*, the development of experimental lines for fine-mapping *SDV1*, mapping of *SDV2*, verification of *SDV2* and survey of gametic distortion. Long and short vertical bars respectively denote chromosomes 4 and 6. Blue and pink segments respectively depict chromosomal segments from *O. sativa* and *O. meridionalis*. The chomosomal locations of *SDV1* and *SDV2* are indicated by horizontal bars on the chromosomes. The number in parenthesis indicates the segregating genes: 1 and 2 denote *SDV1* and *SDV2*, respectively. Dotted X mark indicates that the genotype is aborted during seed development.

Cultivated rice (*Oryza sativa*) is not only a staple food for many people, but is also an extremely important grain from a food security perspective. In the process of rice breeding, AA genome wild rice is considered an effective gene pool because it is closely related to cultivated rice and is highly diverse (Brozynska *et al.* 2016, Henry *et al.* 2010). However, reproductive barriers often occur in crosses between cultivated and wild rice. There have been numerous reports of reproductive barriers in crosses between *O. sativa* and its closely related wild species, and these reports provide very important information for rice breeding and for understanding the mechanisms of reproductive barriers. *Oryza meridionalis* is a wild rice with an AA genome native to Australia, offering a unique evolutionary path compared to other AA genome species (Brozynska *et al.* 2017, Cheng *et al.* 2002, Stein *et al.* 2018, Zhang *et al.* 2014). Reproductive barriers have been reported in a number of crosses between *O. meridionalis* and other AA genomic species (Banaticla-Hilario *et al.* 2013, Ichitani *et al.* 2022, Juliano *et al.* 2005, Li *et al.* 2018, Naredo *et al.* 1997, 1998). In our previous study, we observed that no homozygote of *O. meridionalis* alleles for the loci closely linked with *HD1* on chromosome 6 appeared in progenies from crosses between *O. meridionalis* and *O. sativa* (Toyomoto *et al.* 2019). This was caused by abortion during seed development, indicating that a gene controlling seed development (*SEED DEVELOPMENT 1*, *SDV1*) was located on chromosome 6. *O. sativa* carries the dominant functional fertile allele *Sdv1-s* (*s* stands for *sativa*). *O. meridionalis* is homozygous for the recessive abortive allele *sdv1-m* (*m* stands for *meridionalis*) (Fig. 1). However, *O. meridionalis* is able to self-fertilize and produce seeds, suggesting that it carries a functional fertile gene at other loci probably deriving from gene duplication. A putative duplicative gene of *SDV1*, tentatively named *SEED DEVELOPMENT 2* (*SDV2*), was proposed (Toyomoto *et al.* 2019): *O. meridionalis* and *O. sativa* should carry the functional allele and the nonfunctional allele at the *SDV2* locus, respectively. We named these respective alleles *Sdv2-m* and *sdv2-s* (Fig. 1).

The existence of the *SDV2* gene has remained untested. Seed development is an essential phenomenon for all sexual propagative plant species: All *Oryza* species should carry at least one functional allele of the *SDV1* or *SDV2* locus. AA genome genus *Oryza* comprises seven species, *O. sativa*, *O. glaberrima*, *O. barthii*, *O. rufipogon*, *O. glumaepatula*, *O. meridionalis*, and *O. longistaminata*. Annual accessions of *O. rufipogon* are referred to as *O. nivara* by some researchers (Stein *et al.* 2018, Zhang *et al.* 2014). *O. rufipogon* is the progenitor of *O. sativa*, and *O. barthii* is the progenitor of African rice *O. glaberrima*. The history of duplication and loss of function of *SDV1* and *SDV2* will shed light on the controversial AA-genome *Oryza* speciation (Htet *et al.* 2022, Ohtsubo *et al.* 2004, Stein *et al.* 2018, Yin *et al.* 2015, Zhang *et al.* 2014). In this study, we first performed fine mapping and haplotype analysis of *SDV1*. Then we performed linkage analysis of *SDV2*. Finally, we tested if the two genes follow duplicate gene action, and searched for their candidate genes, utilizing their chromosomal locations to determine how these genes cause reproductive barriers and the genetic basis of these genes.

## Materials and Methods

### Plant material

In this study, we used an *O. meridionalis* accession W1297 and a rice cultivar ‘Taichung 65’ (T65) for linkage analysis of *SDV1* and *SDV2*. W1297 was collected in Darwin, Northern Territory, Australia, and was provided by the National Institute of Genetics in Mishima, Japan. *O. sativa* comprises two subspecies, *japonica* and *indica*. T65 is a *japonica* cultivar used frequently in the study of rice genetics as a recurrent parent of chromosome segment substitution lines, near-isogenic lines, and the study of induced mutation (Ichitani *et al.* 2014, Sano 1990, Yamagata *et al.* 2019, Yoshimura *et al.* 2010). Using T65 genetic background with incorporated W1297 chromosomes, we developed several experimental lines for analyzing *SDV1* and *SDV2* genes. To avoid confusion, number(s) in parenthesis was added after the general generation name to refer to target gene(s), as shown in Fig. 1: 1 and 2 denote *SDV1* and *SDV2*, respectively.

Cultivation was conducted according to the methods described by Ichitani *et al.* (2014). The germinated seeds were sown in seedling boxes in a greenhouse. Approximately two weeks after sowing, the seedlings were transferred out of the greenhouse. Approximately 30 days after sowing, the seedlings were transplanted to the paddy fields in Experimental Farm of the Faculty of Agriculture, Kagoshima University. The applied fertilizers were 4, 6, and 5 g/m^2^, respectively, for N, P_2_O_5_, and K_2_O. The plant spacing was 15 × 30 cm. Seeds were sown in May to June, and seedling transplanting to paddy fields were conducted in late June to July from 2017 to 2023.

For the haplotype analysis, we used nineteen accessions of *O. meridionalis*, four accessions of *Oryza rufipogon* from New Guinea, two accessions of *O. rufipogon* from Australia, fourteen accessions of *O. rufipogon* from Asia, and nine accessions of *O. sativa* (Table 1). All the wild rice accessions except Jpn1 and Jpn2 were distributed from the National Institute of Genetics supported by the National Bioresource Project, MEXT, Japan. Jpn1 and Jpn2 were collected in Australia with the permission from the Queensland government, under the EcoAccess program (Sotowa *et al.* 2013). World Rice Core collection (WRC) by Kojima *et al.* (2005) comprises 69 accessions based on a genome-wide RFLP polymorphism survey of 332 accessions of *O. sativa*. A total of eight WRC lines were selected so that they cover the six groups classified by 40 Indel markers (Ichitani *et al.* 2016).

### Trait evaluation

Seed fertility was evaluated by collecting 50 seeds from the upper side of each of three panicles, following Toyomoto *et al.* (2019). Seemingly sterile seeds were dehusked to see if sterility occurred before or after fertilization.

### DNA analysis

DNA extraction from leaves was performed according to Toyomoto *et al.* (2019). PCR mixtures, cycles, electrophoresis, DNA staining, and gel image recordings were conducted according to Ichitani *et al.* (2014). Antmap (Iwata and Ninomiya 2006) was used to draw the linkage map of *SDV2*. The Kosambi function was used to calculate the map distance.

### DNA markers

Most published PCR-based DNA markers for *Oryza* species are based on an *O. sativa* genome sequence such as ‘Nipponbare’ (Os-Nipponbare-Reference-IRGSP-1.0 (Kawahara *et al.* 2013)) and ‘93-11’ (Yu *et al.* 2002). However, many discrepancies in DNA sequence between the genome of Nipponbare (IRGSP 1.0) and that of *O. meridionalis* accession W2112 (GCA_000338895) (Stein *et al.* 2018) lead to failure in amplification from the *O. meridionalis* genome when using *O. sativa* genome-based DNA markers (Toyomoto *et al.* 2019). Our strategy for designing codominant DNA markers for mapping *SDV1* and *SDV2* genes was to select insertions/deletions (indels) ranging from 5 to 100 base pairs that were shared by the two *O. meridionalis* accessions, W2112 (Stein *et al.* 2018) and IRGC105298 (Zhang *et al.* 2014), and not shared by the following AA genome species sequences, *O. sativa* cv. Nipponbare (Kawahara *et al.* 2013), *O. sativa* cv. Taichung 65 (Wei *et al.* 2016), *O. rufipogon* accession W1943 (Stein *et al.* 2018), *O. nivara* accession IRGC100897 (Stein *et al.* 2018), two *O. longistaminata* accessions W1413 and W1508 (Ohyanagi *et al.* 2016), *O. glumaepatula* accession GEN1233 (Stein *et al.* 2018) and *O. barthii* accession IRGC105608 (Stein *et al.* 2018) with the aid of TASUKE+ (Kumagai *et al.* 2019). Based on sequence similarity surrounding the indels between the Nipponbare (Os-Nipponbare-Reference-IRGSP-1.0) and *O. meridionalis* W2112 (GCA_000338895.3) genome assemblies, the selected indels were further screened. Primer design followed Busungu *et al.* (2016). Mismatches between primer sequences and genomic sequences were checked using Oryzabase BLAST (https://shigen.nig.ac.jp/rice/oryzabase/locale/change?lang=en) (Yamazaki *et al.* 2010) and MBKBASE BLAST (Peng *et al.* 2020). A single base mismatch within the primer sequence was accepted.

## Results

### Fine mapping of *SDV1*

Our previous study showed that *SDV1* is located between two indel markers, KGC6_10.09 and KGC6_22.19 (approximately 12 Mb) (Toyomoto *et al.* 2019). To narrow down the candidate region for *SDV1*, we first selected 115 recombinants between the two DNA markers KGC6_10.09 and KGC6_22.19 from 4625 individuals in a BC_4_F_4_(1) population. Four plants were recombinants between KGC6_10.09 and KGC6_13.00. Next, we designed 10 new DNA markers located between the two DNA markers (Supplemental Table 1), and applied them to the four plants (Table 2). Because homozygotes for W1297 at *SDV1* locus are aborted during seed development (Toyomoto *et al.* 2019), the *SDV1* locus should not be located near any DNA markers for which genotypes were homozygous for W1297 allele in the four plants (Table 2, Fig. 1). The experimental result indicates that the candidate region for the *SDV1* locus was narrowed down to 333 kb encompassed by KGC6_11.53 and KGC6_11.87.

### Haplotype analysis around *SDV1*

The genotypes of 48 accessions for the nine DNA markers located around the *SDV1* locus are presented in Table 1. As described in the Materials and Methods, the DNA markers in Supplemental Table 1 were designed to detect the DNA polymorphism between two *O. meridionalis* accessions and the other AA genome *Oryza* species. All the *O. meridionalis* accessions, W1235 and W1239 shared the same banding patterns of DNA markers located in the candidate *SDV1* region. It has been proposed that the W1235 and W1239 accessions in New Guinea might have been misclassified as *O. rufipogon*, and that they probably are *O. meridionalis* (Htet *et al.* 2022, Yin *et al.* 2015) : these two accessions belonged to a same cluster based on nuclear SSR markers as *O. meridionalis* (Yin *et al.* 2015), and shared a large intron in *PolA1* gene with *O. meridionalis* (Htet *et al.* 2022). These results suggest that *O. meridionalis* accessions in Australia and New Guinea share the *sdv1-m* allele in common, though *O. meridionalis* and *O. rufipogon* accessions in Australia share chloroplast DNA polymorphisms (Sotowa *et al.* 2013), and they share the same habitats in Australia (Henry 2019).

### Mapping of the candidate of *SDV2*

Because segregation distortion and seed abortion caused by *sdv1-m* were observed in the T65 genetic background, those caused by *sdv2-s* should be observed when the *SDV1* locus is fixed for *sdv1-m*. We therefore developed experimental lines for mapping *SDV2* gene (Fig. 1). Among thirty nine BC_1_F_1_ plants, ten latest heading plants were selected: they were expected to be heterozygote for *SDV*1 locus because *SDV1* is closely linked with *HD1* locus, which mainly controlled heading date in the progeny from the cross between T65 and W1297, and W1297 carries late heading allele at *HD1* (Toyomoto *et al.* 2019). Approximately 80 BC_1_F_2_(2) plants for each BC_1_F_1_ plant were genotyped for the DNA markers linked with *SDV1*. The eight BC_1_F_2_(2) lines showed segregation distortion of the DNA markers explainable by *SDV1* gene segregation: no homozygotes of W1297 allele at KGC6_12.02 appeared. This suggests that they became homozygous for *sdv2-s* by one cycle of backcrossing. One BC_1_F_1_ plant proved to be recombinant between *HD1* and *SDV1*: it was fixed for T65 allele of the DNA markers linked with *SDV1* gene. The BC_1_F_2_(2) from one BC_1_F_1_ plant showed the segregation of 28 T65 homozygotes: 40 heterozygotes: 9 W1297 homozygotes at KGC6_12.02 locus, skewed from the expected Mendelian ratio 1:2:1 (*χ*^2^ = 9.493, *P* = 0.009). However, the existence of W1297 homozygotes suggests that *SDV2* gene could be also segregating in this population. Among 9 BC_1_F_2_(2) plants homozygous of W1297 allele at KGC6_12.02 locus, four semi-sterile BC_1_F_2_(2) plants were selected by visual observation of seed fertility (Fig. 1). This selection was based on the idea that highly fertile plants were expected to be fixed for *Sdv2-m* allele for *SDV2* locus, and highly sterile plants were expected to be segregating for other loci affecting seed fertility. BC_1_F_3_(2) in Fig. 1 were subjected to further genetic analysis. The bulked DNA from ten plants from each BC_1_F_3_(2) lines were genotyped for 80 DNA markers covering all the 12 rice chromosomes: no homozygotes of T65 alleles were detected for some DNA markers on chromosomes 4, 5, and 8. BC_1_F_4_(2) lines derived from BC_1_F_3_(2) plants heterozygous for these markers were subjected to a preliminary linkage analysis with a small number of plants, showing that *SDV2* could be located on chromosome 4. Then we repeatedly selected possible heterozygotes of the *SDV2* from BC_1_F_4_(2) to BC_1_F_6_(2) generation with the aid of DNA markers and seed fertility data. The repeated selfing process could make the other loci homozygous, which was expected to reduce the variance of seed fertility caused by genes other than *SDV2*.

**Fig. 2.**
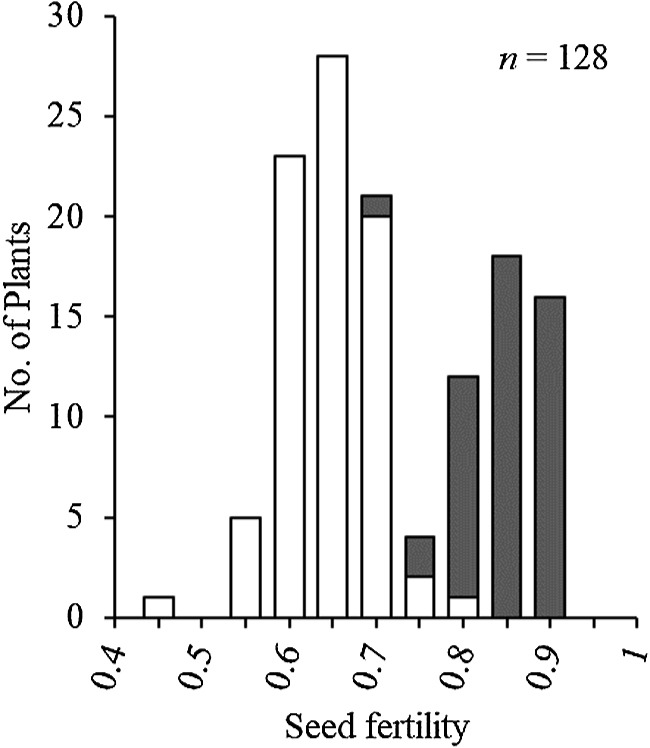
Frequency distribution of seed fertility of BC_1_F_7_(2) population. Two classified genotypes were assessed for KGC4_21.00 as indicated: *grey*, homozygotes for W1297; and *white*, heterozygotes. No homozygotes for T65 appeared in this population.

The BC_1_F_7_(2) generation showed a bimodal distribution of seed fertility (Fig. 2). No homozygotes of T65 allele were present in consecutive DNA markers from KGC4_19.55 to KGC4_21.33, supporting that *SDV2* candidate gene is linked with these DNA markers (Supplemental Table 2). The four DNA markers, KGC4_20.55, KGC4_20.67, KGC4_21.00, and KGC4_21.33 cosegregated with one another. The genotypes of these four markers explained the variation of seed fertility: The homozygotes of W1297 allele were distributed toward high seed fertility, and the heterozygotes were distributed toward low seed fertility (Fig. 2). Several recombinants between the four DNA marker set and the other DNA markers were subjected to a progeny test to determine if homozygotes of T65 allele appeared or not (Table 3): All the recombinants supported cosegregation of the candidate *SDV2* with the four DNA markers. In the BC_1_F_7_(2) generation, the segregation ratio of W1297 homozygote: heterozygote: T65 homozygote is 49:80:0, which is fitted to the expected ratio 1:2:0, the gene model that homozygotes of T65 allele at *SDV2* locus are aborted (Fig. 1).

**Fig. 3.**
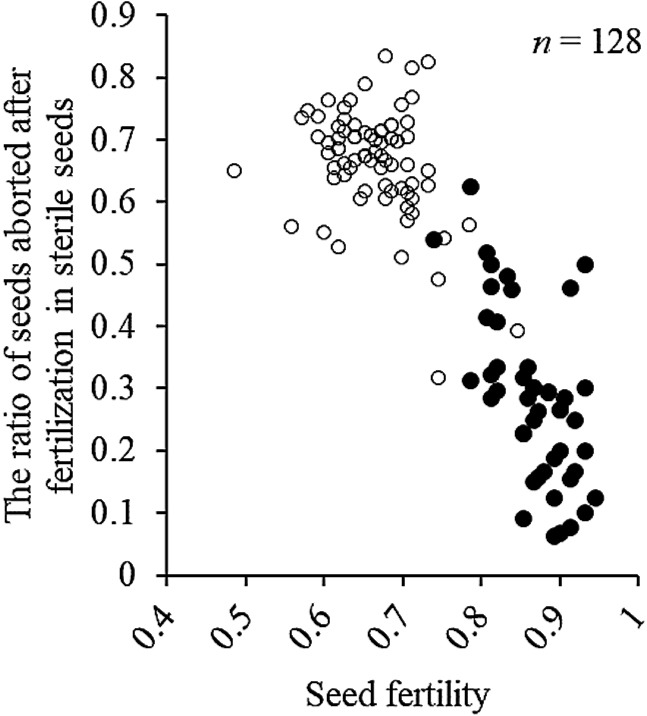
Scatter diagram between seed fertility (the ratio of fertile seeds) and the ratio of seeds aborted after fertilization in BC_1_F_7_(2) population. Two classified genotypes were assessed for KGC4_21.00 as indicated: *solid circles*, homozygotes for W1297; and *open circles*, heterozygotes.

If the *SDV2* gene is the counterpart of *SDV1* gene, lower fertility of heterozygotes than homozygotes of W1297 allele at *SDV2* locus can be explained by abortion after fertilization, the same gene model as *SDV1* (Toyomoto *et al.* 2019). Sterile seeds of BC_1_F_7_(2) plants were dehusked to see if abortion occurred before or after fertilization. The proportion of seeds aborted after fertilization for heterozygotes for KGC4_21.00 was higher than that for homozygotes of the W1297 allele (Fig. 3). This experimental result also supports that *SDV2* is located on chromosome 4, closely linked with the DNA marker KGC4_21.00.

**Fig. 4.**
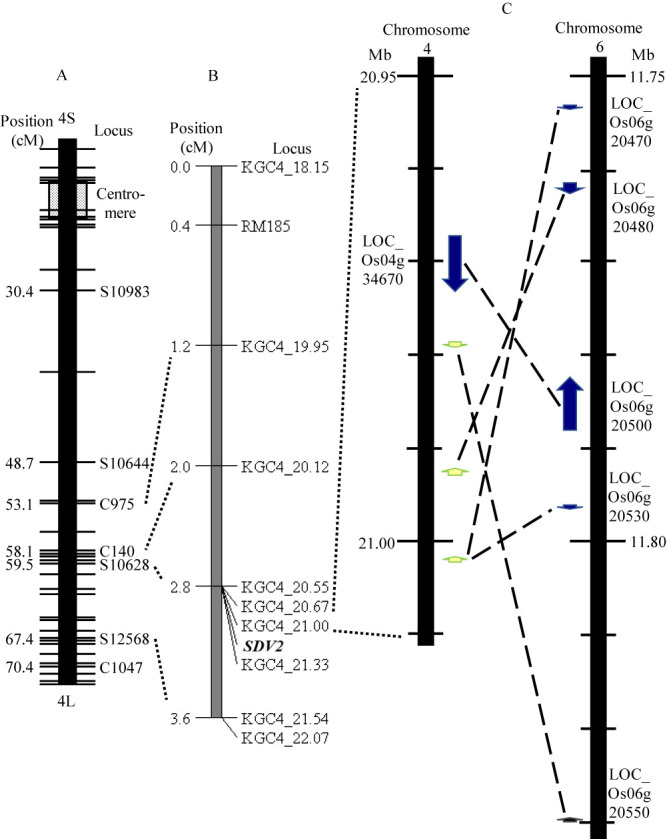
Linkage map showing *SDV2* gene on the long arm of chromosome 4. A: RFLP framework map of chromosome 4 modified from Harushima *et al.* (1998). B: Linkage map of the *SDV2* gene constructed from the BC_1_F_7_(2) population (*n* = 128). C: The comparative physical map of the duplicated region of chromosomes 4 and 6. Blue arrows indicate genes annotated by Rice Genome Annotation Project. LOC_Os06g20500 and LOC_Os04g34670 are paralogous genes to each other. LOC_Os06g20470 and LOC_Os06g20530 are also paralogous genes to each other. Green arrows indicate DNA sequences on chromosome 4 homologous to the genes on chromosome 6. The direction of the arrowhead indicates the strand of DNA sequences shown in Supplemental Table 3. DNA markers, genes and/or DNA sequences located near each other on Nipponbare pseudomolecules are connected by dotted lines. Homologous DNA sequences to each other on chromosomes 4 and 6 are connected by long dashed lines.

Based on the inference that the genotype of candidate *SDV2* gene was the same as KGC4_21.00, a linkage map of candidate *SDV2* gene was constructed (Fig. 4). The candidate *SDV2* gene is located on the long arm of chromosome 4.

### Verification of *SDV2*

**Fig. 5.**
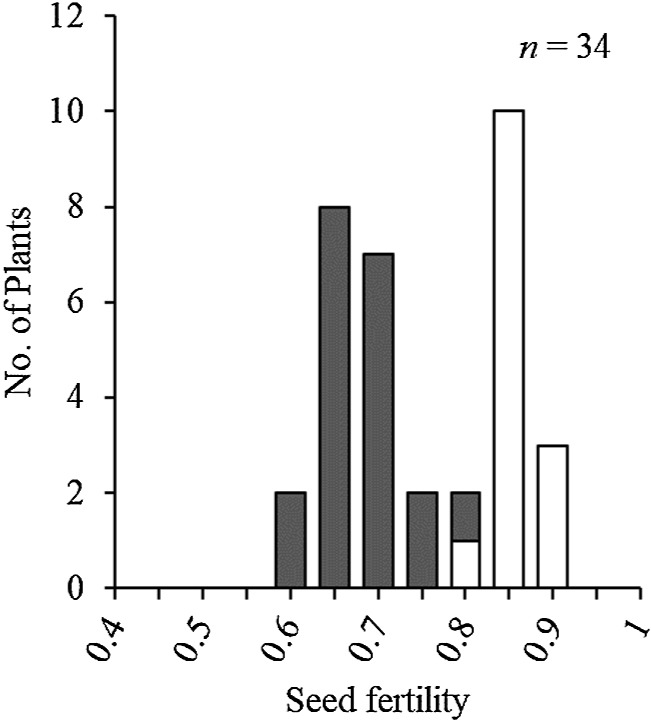
Frequency distribution of seed fertility of BC_2_F_1_(2) population. Two classified genotypes were assessed for KGC4_21.00 as indicated: *grey*, homozygotes for T65; and *white*, heterozygotes.

If the gene closely links with the above four DNA markers on chromosome 4 is the true *SDV2*, the combination of *sdv1-m*/*sdv1-m*, the homozygote of W1297 allele at *SDV1* locus, and *sdv2-s*/*sdv2-s*, the homozygote of T65 allele at *SDV2* locus, should not be found in a population in which both *SDV1* and *SDV2* genes are segregating (Fig. 1). We developed such experimental population: a heterozygote at candidate *SDV2* locus was selected with the aid of DNA marker in a BC_1_F_5_(2) population, and was backcrossed to T65 as pollen donor to develop BC_2_F_1_(1, 2) population. This population showed a bimodal distribution of seed fertility, which is explainable by the genotype of KGC4_21.00, a DNA marker cosegregating with candidate *SDV2* (Fig. 5). This population shared the genotype *Sdv1-s*/*sdv1-m* (Fig. 1). The fact that homozygotes of T65 allele at KGC4_21.00 showed low seed fertility, and heterozygotes showed high seed fertility supports our genetic model.

Then we examined the BC_2_F_2_(1, 2) population derived from BC_2_F_1_(1, 2) plants heterozygous both at KGC6_11.74, a cosegregating DNA marker of *SDV1*, and KGC4_21.00, a cosegregating DNA marker of candidate *SDV2*. The combination of genotypes of the both DNA markers of 1,373 plants were examined. Among the possible nine genotype combinations, the homozygote of W1297 allele at KGC6_11.74 combined with the homozygote of T65 allele at KGC4_21.00 did not exist (Table 4, Fig. 1). This result verifies that *SDV2* gene is tightly linked with a DNA marker KGC4_21.00 located on chromosome 4.

The segregation ratio of KGC4_21.00 were highly distorted in all the three genotypes at the KGC6_11.74 locus (Table 4). The distortion of KGC4_21.00 under homozygotes of T65 allele and heterozygotes at the KGC6_11.74 locus cannot be explained by the combination of *SDV1* and *SDV2* genes. A BC_3_F_1_(1, 2) plant heterozygous at KGC6_11.74 and KGC4_21.00 was reciprocally backcrossed to T65 to develop a BC_4_F_1_(1, 2) generation (Fig. 1). When T65 was used as the egg donor, the genotype ratio of KGC4_21.00 deviated significantly from the expected ratio 1:1 (Table 5). This suggests that segregation distortion was caused by a gene causing male gamete abortion linked with *SDV2*: pollen carrying the W1297 allele were aborted (Table 5). The combination of the two genotypes KGC6_11.74 and KGC4_21.00 fitted to the ratio assuming their independent inheritance for reciprocal crosses (Table 5). If *SDV1* and *SDV2* gametophyically interacted to cause pollen sterility or egg sterility, the ratio of plants with heterozygotes for KCG4_21.00 and homozygotes for KGC6_11.74 or plants with heterozygotes for KGC6_11.74 and homozygotes for KCG4_21.00 would be reduced from the expected ratio assuming their independent inheritance, as seen in *DPL1* and *DPL2* genes (Mizuta *et al.* 2010). Explanation of these genes are in Discussion section. The independent inheritance of KGC6_11.74 and KCG4_21.00 suggests that the genes on *SDV1* and *SDV2* do not interact gametophytically.

### Search for candidate gene of *SDV1*

We narrowed down the chromosomal location of *SDV1* to approximately 333 kbp between the DNA markers KGC6_11.53 and KGC6_11.87. According to Rice Genome Annotation Project (http://rice.uga.edu/) (Kawahara *et al.* 2013), this region is predicted to contain 30 protein-coding genes (Table 6). BLAST search for orthologous genes in *O. meridionalis* genome was performed using MEGABLAST optimized for highly similar sequences (https://blast.ncbi.nlm.nih.gov/Blast.cgi), with the genomic sequence of predicted genes used as query, and the genomic sequence *O. meridionalis* accession W2112 (coded GCA_000338895.3) used as subject. Most of the genes ranging from LOC_Os06g20130 to LOC_Os06g20430, and LOC_Os06g20570 and LOC_Os06g20610 have conserved DNA sequences in chromosome 6 of W2112 in the same order. The four genes, LOC_Os06g20470, LOC_Os06g20500, LOC_Os06g20530 and LOC_Os06g20550, have conserved DNA sequences in the candidate chromosomal region of *SDV2* gene on chromosome 4, not on chromosome 6 of W2112 (Table 6, Supplemental Table 2). These results suggest that these four genes could be candidate genes for *SDV1*, and that their orthologous genes located on chromosome 4 of W2112 could be the candidate genes for *SDV2*.

We further examined the candidate chromosomal region of *SDV1*, using the genome sequences of eight AA genome *Oryza* species (Supplemental Tables 3, 4). We found that the each of the five genes, LOC_Os06g20470, LOC_Os06g20480, LOC_Os06g20500, LOC_Os06g20530 and LOC_Os06g20550, were duplicated and located nearby on chromosomes 6, and also located on chromosome 4 with a few exceptions in the three species, *O. sativa*, *O. rufipogon* and *O. nivara* (Supplemental Table 3). LOC_Os06g20470 and LOC_Os06g20530 are examples of a pair of duplicate genes on chromosome 6 (Fig. 4, Supplemental Table 3). The paralogous sequences of *O. sativa* on chromosome 4 are located within *SDV2* candidate region (Fig. 4, Supplemental Table 3). On the other hand, the orthologous sequences of the four species, *O. meridionalis*, *O. glaberrima*, *O. barthii* and *O. glumaepatula*, are located only on chromosome 4. The order and orientation of the conserved gene sequences on chromosome 4 are shared by all eight species except that *O. longistaminata* shares the order and orientation of the above orthologous sequences with the other seven species on chromosome 11 (Supplemental Table 3).

The five genes located upstream and downstream of the duplicated region in Supplemental Table 3 were selected because they are single copy genes in *O. sativa*, and histories of their orthologous genes could be discussed simply. The seven species, *O. sativa*, *O. rufipogon*, *O. nivara*, *O. meridionalis*, *O. glaberrima*, *O. barthii*, and *O. glumaepatula*, share the same orders and orientations on the five upstream genes on chromosome 6. *O. longistaminata* carries these genes in the opposite orientation and order on chromosome 3. As for the five downstream genes, the all eight AA genome *Oryza* species share them in the same order and orientation on chromosome 6.

These data strongly suggest that the duplication and loss of the candidate chromosomal region of *SDV1* of an *O. sativa* cultivar Nipponbare and *O. meridionalis* accession W2112 are not confined to the two accessions but can be extended to the history of AA genome *Oryza* species. They also support the above inference that the five genes, LOC_Os06g20470, LOC_Os06g20480, LOC_Os06g20500, LOC_Os06g20530 and LOC_Os06g20550, are the candidate *SDV1* genes. Among them, only LOC_Os06g20500 is predicted to have a specific function described as tRNA-splicing endonuclease positive effector-related, putative, expressed, and the other four genes are described to encode expressed proteins. According to Rice Genome Annotation Project (http://rice.uga.edu/) (Kawahara *et al.* 2013), LOC_Os06g20500 is expressed in all ten tissues including seed-5 days after pollination (DAP), seed-10 DAP, embryo-25 DAP and endosperm-25 DAP. On the other hand, the other four candidate genes are not expressed in these tissues.

## Discussion

In this study, we narrowed down the chromosomal location of *SDV1* to approximately 333 kbp between the DNA markers KGK KG C6_11.53 and KG C6_11.87 on chromosome 6. The chromosomal region is highly conserved among many *O. meridionalis* accessions, which is consistent with linkage analysis of *SDV1*. We also performed linkage analysis of the candidate of *SDV2*, and found that it is located between KGC4_20.12 and KGC4_21.54, co-segregating with KGC4_20.55, KGC4_20.67, KGC4_21.00 and KGC4_21.33 on chromosome 4. The candidate chromosomal location of *SDV2* proved to be correct in an experiment using a population in which both *SDV1* and *SDV2* genes were segregating. The chromosomal region covering *SDV1* gene was predicted to contain 30 protein-coding genes in *O. sativa*. Among them, five genes had conserved DNA sequences in the candidate chromosomal region of *SDV2* gene on chromosome 4, not on chromosome 6, of the genome of an *O. meridionalis* accession W2112. These results suggest that these five genes could be candidate genes for *SDV1*, and that their orthologous genes located on chromosome 4 of W2112 could be candidate genes for *SDV2*. The gene expression data suggested that the candidate can be confined to LOC_Os06g20500. However, the gene has two paralogous genes in the rice genome Os-Nipponbare-Reference-IRGSP-1.0, LOC_Os04g34670 located on the candidate *SDV2* region of chromosome 4 and LOC_Os03g32526 located on chromosome 3. They are also expressed in in all ten tissues including seed-5 days after pollination (DAP), seed-10 DAP, embryo-25 DAP and endosperm-25 DAP. Further linkage analysis on *SDV1* and *SDV2*, DNA sequence comparison of candidate chromosomal regions between chromosomes 4 and 6 among AA genome *Oryza* species and fine-tuned gene expression study targeting the above candidate genes will contribute to identification of *SDV1* and *SDV2* genes.

**Fig. 6.**
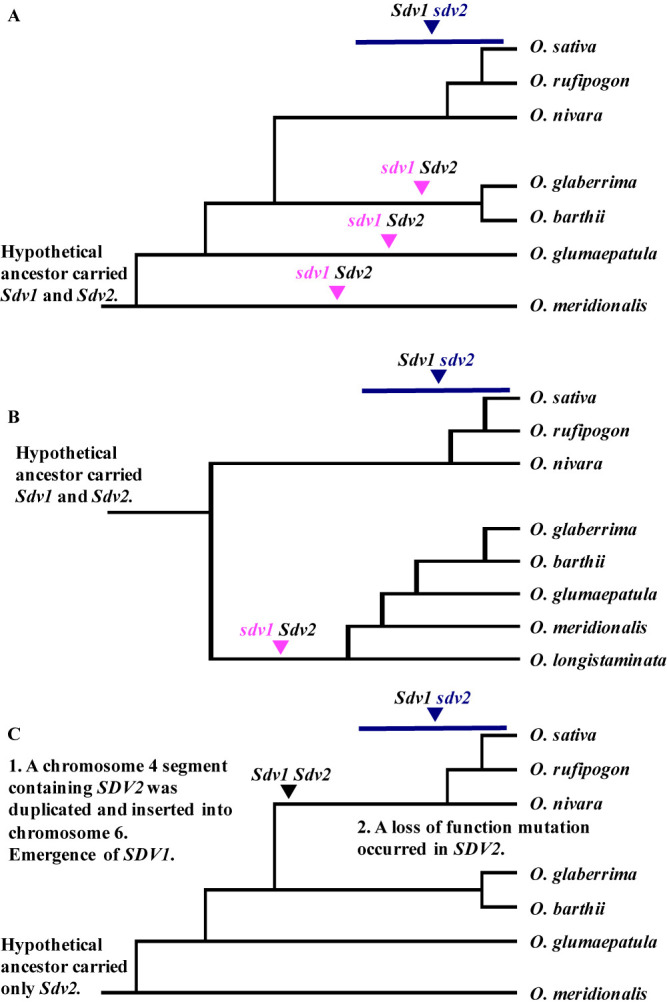
Working hypotheses for the evolution of functionally duplicate genes, *SDV1* and *SDV2*, in AA genome *Oryza* species. A, a model based on phylogeny from genomic data by Zhang *et al.* (2014) and Stein *et al.* (2018), assuming that the hypothetical ancestor carried *Sdv1*, the functional allele at *SDV1* locus, and *Sdv2*, the functional allele at the *SDV2* locus. Mutation from *Sdv1* to the abortive allele *sdv1* including gene loss at the *SDV1* locus occurred once, and that from *Sdv2* to the abortive allele *sdv2* on *SDV2* locus occurred at least three times. *O. longistaminata* is excluded because Zhang *et al.* (2014) and Stein *et al.* (2018) did not test this species. B, a model assuming least mutations of the *SDV1* and *SDV2* loci, and that the hypothetical ancestor carried both *Sdv1* and *Sdv2*. The above mutation event occurred once at each locus. C, a model based on phylogeny from genomic data by Zhang *et al.* (2014) and Stein *et al.* (2018), assuming that the hypothetical ancestor carried only *Sdv2*. The duplication of *SDV2* turning into *SDV1* and loss of function of the original *SDV2* occurred in the common ancestor of *O. sativa*, *O. rufipogon* and *O. nivara* after divergence from the common ancestor of *O. glaberrima* and *O. barthii*.

Based on Supplemental Table 3, three phylogenetic trees of the eight species can be drawn (Fig. 6). Because the *Oryza* genome has undergone whole genome duplication (Salse *et al.* 2008, Yu *et al.* 2005), we first assumed that hypothetical ancestor carried functional *SDV1* and *SDV2* genes (Fig. 6). Fig. 6A reflects phylogeny from genomic data by Zhang *et al.* (2014) and Stein *et al.* (2018). At least three mutation events including gene loss on the *SDV1* locus and one mutation on the *SDV2* occurred in this model. Mizuta *et al.* (2010) detected paralogous hybrid incompatibility genes, *DOPPELGANGER1* (*DPL1*) and *DOPPELGANGER2* (*DPL2*), cause loss of germination of pollen in intraspecific cross in *O. sativa*. Independent disruptions of *DPL1* and *DPL2* occurred in *indica* and *japonica*, respectively. Pollen carrying two defective *DPL* alleles became nonfunctional and did not germinate, suggesting an essential role for *DPL*s in pollen germination. *DPL1* corresponds to LOC_Os01g15448 located at 8.65 Mb on chromosome 1, and *DPL2* gene corresponds to LOC_Os06g08510 located at 4.20 Mb on chromosome 6. Loss-of-function mutations of *DPL1* genes emerged multiple times in *indica* and its wild ancestor *O. rufipogon*, and the *DPL2* gene defect is specific to *japonica* cultivars. Bikard *et al.* (2009) showed that in a cross between two *Arabidopsis thaliana* accessions Columbia-0 and Cape Verde Island accession Cvi-0, the *LD1.1* locus on chromosome 1 and *LD1.5* locus on chromosome 5 interact epistatically to control recessive embryonic lethality, and this was explained by divergent evolution between paralogs of essential duplicated genes. This genetic model is same as that of *SDV1* and *SDV2*. These reports suggest that Fig. 6A assuming multiple mutations is not improbable. Fig. 6B assumes the least number of mutations at the *SDV1* and *SDV2* loci: The above mutation event occurred once at each locus. This tree is different from Fig. 6A reflecting the evolution of the whole genome. It seems improbable that duplication and loss of *SDV1* and *SDV2* genes occurred independent of the evolution of the whole genome. Fig. 6C assumes that the hypothetical ancestor carried only the functional *SDV2* gene, and that duplication of *SDV2* turning into *SDV1* and loss of function of the original *SDV2* gene occurred in the common ancestor of *O. sativa*, *O. rufipogon* and *O. nivara* after divergence from the common ancestor of *O. glaberrima* and *O. barthii*. Remarkably it seems that both gene duplication and loss occurred in one linage in an evolutionarily short period. However, this hypothesis is consistent with the evolution of the whole genome, and assumes the least mutation events. Further investigation of *SDV*s would deepen our knowledge of the duplication phenomenon and speciation.

There have been many reports of hybrid sterility genes found in interspecific crosses among AA genome *Oryza* species located close to *SDV1* and *SDV2*. Supplemental Table 5 shows isolated or DNA-marker tagged genes causing sterility found in distant crosses located on chromosomes 4 and 6. Among the genes above, *Cif_2_*, *Su-Cif* and *cim* are located close to *HD1* (Koide *et al.* 2008, Matsubara *et al.* 2003), hence *SDV1*, on chromosome 6. Matsubara *et al.* (2003) reported unidirectional cross-incompatibility detected in advanced generation of backcrossing between *O. rufipogon* accessions and *O. sativa* accessions. The near-isogenic line of the line named T65*wx* (*japonica* type) carrying an alien segment of chromosome 6 from *O. rufipogon* gave a reduced seed setting only when crossed with T65*wx* as the male. The four causal genes, *Cif_1_*, *Cif_2_*, *cim* and *Su-Cif*, are involved in this phenomenon (Koide *et al.* 2008). This unidirectional cross-incompatibility controlled by these genes occurred during the development of hybrid seeds. Though the morphology and gene location are similar between the unidirectional cross-incompatibility (Matsubara *et al.* 2003) and seed abortion by *SDV1* and *SDV2* genes, their genetic models are different: homozygotes of loss of function genes can not develop into mature seeds in our model. The location of *S6* is close to that of *SDV1*. However, these genes are distinct judging from the gene function.

As for the genes on chromosome 4, locations of *S9* and *DUPLICATED GAMETOPHYTIC STERILITY 1* (*DGS1*) are close to that of *SDV2*. *S9* was detected in intraspecific cross among *O. sativa* (Zhao *et al.* 2006), causing female gamete abortion. *DGS1* gene was detected in the cross between *O. sativa* and *O. nivara* (Nguyen *et al.* 2017). Pollen fertility was controlled by gametophytic gene combination on *DGS1* and *DUPLICATED GAMETOPHYTIC STERILITY 2* (*DGS2*) loci: pollen carrying *O. nivara* allele at *DGS1* and *O. sativa* allele at *DGS2* is sterile. *DGS1* is located on chromosome 4, and *DGS2* is located on chromosome 7. The two genes encode protein homologous to DNA-dependent RNA polymerase (RNAP) III subunit C4 (RPC4). The combination of loss of the functional allele at *DGS1* and *DGS2* caused hybrid pollen sterility gametophytically. However, *S9* and *DGS1* were distinct from *SDV2*, judging from the gene segregation pattern.

In BC_2_F_2_(1, 2) population and BC_4_F_1_(1, 2) generation using T65 as egg donor, genotypes of KGC4_21.00 was significantly skewed from the expected Mendelian ratio (Tables 4, 5). No gametophytic interaction genes linked with KGC4_21.00 and KGC6_11.74 was observed. These data suggest that a gene causing male gamete abortion carrying the allele from W1297 is linked with *SDV2*. Such a gene might have been fixed in BC_1_F_7_(2) for mapping *SDV2* (Fig. 1). The recombination value of the gene and KGC4_21.00 is roughly estimated to be 0.128 (6/47). The chromosomal locations of *SDV2* (Supplemental Table 2, Fig. 4), *S9* (Zhao *et al.* 2006) and *DGS1* (Nguyen *et al.* 2017) (Supplemental Table 5) suggest that the gene causing male gamete abortion is different from *S9* and *DGS1*.

Isolation of *SDV1* and *SDV2* genes will contribute to elucidation of speciation of AA genome *Oryza* species, monitoring possible ongoing hybridization between *O. rufipogon* and *O. meridionalis* in Australia, molecular biology of seed development and rice breeding: Useful genes located close to *sdv1-m* gene on chromosome 6 in *O. meridionalis* genome will be efficiently incorporated into *O. sativa* genome in combination with *Sdv2-m* on chromosome 4. Supplemental Table 3 suggests that this strategy could be applied to using *O. barthii*, *O. glaberrima* and *O. glumaepatula* as useful gene donors.

## Author Contribution Statement

K.I. conceptualized and developed the methodology. D.T., Y.S., and M.U. validated the research. D.T., Y.S., M.U., and K.I. conducted the formal analysis and investigation. S.T., T.S., R.H., R.I., and K.I. provided the resources. D.T., Y.S., M.U., and K.I. curated the data. D.T. and K.I. prepared the original draft. D.T., M.U., S.T., T.S., R.H., R.I., and K.I. reviewed and edited the writing. D.T., Y.S., M.U., and K.I. were responsible for the visualization. K.I. supervised the project. K.I. and R.I. administered the project and acquired funding. All authors have read and consented to the published this version of the manuscript.

## Supplementary Material

Supplemental Tables

## Figures and Tables

**Table 1.** Haplotypes of DNA markers around *SDV1* gene

Accession name	Origin	Genotype of DNA marker*^a^*								
		KGC6_11.53	KGC6_11.67	KGC6_11.71	KGC6_11.732	KGC6_11.736	KGC6_11.737	KGC6_11.74	KGC6_11.84	KGC6_11.87
*Oryza meridionalis*										
W1297	Australia	W	W	W	W	W	W	W	W	W
W1299	Australia	W	W	W	W	W	W	W	W	W
W1300	Australia	W	W	W	W	W	W	W	W	W
W1627	Australia	W	W	W	W	W	W	W	W	W
W1631	Australia	W	W	W	W	W	W	W	W	W
W1635	Australia	W	W	W	W	W	W	W	W	W
W1638	Australia	W	W	W	W	W	W	W	W	W
W2069	Australia	W	W	W	W	W	W	W	W	W
W2071	Australia	W	W	W	W	W	W	W	W	W
W2077	Australia	W	W	W	W	W	W	W	W	W
W2079	Australia	W	W	W	W	W	W	W	W	W
W2080	Australia	W	W	W	W	W	W	W	W	W
W2081	Australia	W	W	W	W	W	W	W	W	W
W2100	Australia	W	W	W	W	W	W	W	W	W
W2103	Australia	W	W	W	W	W	W	W	W	W
W2105	Australia	W	W	W	W	W	W	W	W	W
W2112	Australia	W	W	W	W	W	W	W	W	W
W2116	Australia	W	W	W	W	W	W	W	W	W
Jpn2	Australia	W	W	W	W	W	W	W	W	W
*Oryza rufipogon* in Australia										
W2109	Australia	T	T	T	T	T	T	T	T	T
Jpn1	Australia	T	T	T	T	T	T	T	T	T
*Oryza rufipogon* in New Guinea										
W1230	Indonesia	T	T	T	T	T	T	T	T	T
W1235	Indonesia	W	W	W	W	W	W	W	W	W
W1239	Indonesia	W	W	W	W	W	W	W	W	W
W1236	Papua New Guinea	T	T	T	T	T	T	T	T	T
*Oryza rufipogon* in Asian Countries										
W0106	India	T	T	T	T	T	T	T	T	T
W0107	India	T	T	T	T	T	T	T	T	T
W0120	India	T	T	T	T	T	T	T	1	T
W0137	India	N	T	T	T	T	T	T	T	T
W0630	Burma	T	T	T	T	T	T	T	1	T
W1294	Philippines	N	T	T	T	T	T	T	T	T
W1551	Thailand	T	T	T	T	T	T	T	T	T
W1681	India	T	T	T	T	T	T	T	T	T
W1690	Thailand	T	T	T	T	T	T	T	T	T
W1807	Sri Lanka	1	T	T	T	T	T	T	T	T
W1865	Thailand	T	T	T	T	T	T	T	T	T
W1866	Thailand	T	T	T	T	T	T	T	T	T
W1945	China	T	T	T	T	T	T	T	T	T
W2014	India	N	T	T	T	T	T	T	T	T
*Oryza sativa*										
WRC01	Japan	T	T	T	T	T	T	T	T	T
WRC02	India	T	T	T	T	T	T	T	T	T
WRC21	Myanmar	T	T	T	T	T	T	T	T	T
WRC37	India	T	T	T	T	T	T	T	T	T
WRC43	China	T	T	T	T	T	T	T	T	T
WRC50	America	T	T	T	T	T	T	T	T	T
WRC51	Japan	T	T	T	T	T	T	T	T	T
WRC98	China	2	T	T	T	T	T	T	T	T
Taichung 65	Taiwan	T	T	T	T	T	T	T	T	T

*^a^* T and W represent the same banding pattern as T65 and W1297, respectively. Numbers indicate a banding pattern other than that found in T65 and W1297. N indicates that the band did not appear.

**Table 2.** Genotypes of informative recombinants BC_4_F_4_(1) for DNA marker loci closely located with *SDV1*

BC_4_F_4_(1) Recombinant	Genotype at DNA Marker*^a^*													
	KGC6_10.09	KGC6_10.87	KGC6_11.53	KGC6_11.67	KGC6_11.71	KGC6_11.732	KGC6_11.736	KGC6_11.737	KGC6_11.74	KGC6_11.84	KGC6_11.87	KGC6_12.02	KGC6_13.00	KGC6_22.19
1	W	W	H	H	H	H	H	H	H	H	H	H	H	H
2	H	H	H	H	H	H	H	H	H	H	H	H	W	W
3	H	H	H	H	H	H	H	H	H	H	W	W	W	W
4	W	W	W	H	H	H	H	H	H	H	H	H	H	H

*^a^* W and H respectively denote homozygotes for W1297 and heterozygotes.

**Table 3.** Segregation of DNA markers of the progeny of the informative recombinants in BC_1_F_7_(2) around *SDV2* locus

BC_1_F_7_(2) Recombinant	Genotype at the DNA marker*^a^*							Seed Fertility	Segregation in BC_1_F_8_(2) generation*^b^*		
	KGC4_19.95	KGC4_20.12	KGC4_20.55	KGC4_21.00	KGC4_21.33	KGC4_21.54	KGC4_22.07		KGC4_20.12	KGC4_21.00	KGC4_22.07
1	W	W	W	W	W	H	H	0.88			15:19:13
2	H	H	H	H	H	T	T	0.69		10:36:0	
3	W	W	H	H	H	H	H	0.71		10:17:0	
4	W	H	H	H	H	H	H	0.57	17:24:0		

*^a^* W, H, and T respectively denote homozygotes for W1297, heterozygotes and homozygotes for T65. *^b^* Number of homozygotes for W1297, heterozygotes and homozygotes for T65 are shown in this order.

**Table 4.** Segregation of DNA markers KGC4_21.00 and KGC6_11.74 of BC_2_F_2_(1, 2) generation

		Genotype of KGC4_21.00*^a^*			Sum	χ^2^ (1:2:1)
		T	H	W		
Genotypes of KGC6_11.74 *^a^*	T	137	190	70	397	*P* < 0.001
	H	211	384	108	703	*P* < 0.001
	W	0	218	55	273	*P* < 0.001
Sum		348	792	233		
*χ*^2^ (1:2:1)		*P* < 0.001	*P* = 0.258	*P* = 0.205		

*^a^* T, H, and W denote homozygotes for T65, heterozygotes, and homozygotes for W1297, respectively.

**Table 5.** Segregation of BC_4_F_1_(1, 2) at KGC6_11.74 and KGC4_21.00

		T65 as pollen donor					T65 as egg donor			
		KGC4_21.00*^a^*		Sum	*χ*^2^ (1: 1)		KGC4_21.00*^a^*		Sum	*χ^2^* (1: 1)
		T	H				T	H		
KGC6_11.74*^a^*	T	10	11	21			22	4	26	
	H	6	14	20			19	2	21	
	Sum	16	25		*P* = 0.16		41	6		*P* < 0.001
*χ^2^* (1: 1)				*P* = 0.875					*P* = 0.466	
χ^2^ for independence between										
KGC6_11.74 and KGC4_21_00				*P* = 0.248					*P* = 0.550	

*^a^* T and H denote homozygotes for T65 and heterozygotes, respectively.

**Table 6.** Predicted genes located between KGC6_11.53 and KGC6_11.87, and conserved sequences in *O. meridionalis* genome*^a^*

Predicted genes*^b^*				Conserved sequences in *O. meridionalis* genome*^c^*	
Name	Protein	Location		Chromosome	Location
		(kb)*^d^*			(kb)*^a^*
LOC_Os06g20130	expressed protein	11549			
LOC_Os06g20140	aspartic proteinase nepenthesin-1 precursor, putative, expressed	11552		6	11637
LOC_Os06g20150	peroxidase precursor, putative, expressed	11559		6	11645
LOC_Os06g20180	expressed protein	11575		6	11649
LOC_Os06g20190	aspartic proteinase nepenthesin-2 precursor, putative, expressed	11581		6	11658
LOC_Os06g20200	gibberellin receptor GID1L2, putative, expressed	11586			
LOC_Os06g20240	latency associated nuclear antigen, putative, expressed	11619		2	28908
LOC_Os06g20260	expressed protein	11627			
LOC_Os06g20270	hypothetical protein	11631			
LOC_Os06g20300	expressed protein	11657		6	11680
LOC_Os06g20310	expressed protein	11673		6	11753
LOC_Os06g20320	peptidyl-prolyl cis-trans isomerase, FKBP-type, putative, expressed	11676		6	11761
LOC_Os06g20330	hypothetical protein	11683			
LOC_Os06g20340	dual specificity protein phosphatase, putative, expressed	11687		6	11769
LOC_Os06g20354	PPR repeat domain containing protein, putative, expressed	11693		6	11771
LOC_Os06g20370	microtubule associated protein, putative, expressed	11701		6	11779
LOC_Os06g20380	expressed protein	11706			
LOC_Os06g20390	expressed protein	11712		6	11786
LOC_Os06g20400	DHHC zinc finger domain containing protein, expressed	11722		6	11796
LOC_Os06g20410	BAH domain containing protein, expressed	11734		6	11807
LOC_Os06g20420	expressed protein	11734			
LOC_Os06g20430	BPI/LBP family protein At3g20270 precursor, putative, expressed	11741		6	11816
LOC_Os06g20450	hypothetical protein	11747		8	17376
LOC_Os06g20470	expressed protein	11752		4	19942
LOC_Os06g20480	expressed protein	11761			
LOC_Os06g20500	tRNA-splicing endonuclease positive effector-related, putative, expressed	11787		4	19891
LOC_Os06g20530	expressed protein	11795		4	19942
LOC_Os06g20550	expressed protein	11829		4	19900
LOC_Os06g20570	glycosyltransferase, putative, expressed	11844		6	11823
LOC_Os06g20610	seven in absentia protein family domain containing protein, expressed	11867		6	11835

*^a^* The sequence of *O. meridionalis* accession W2112 (GCA_000338895.3). The detailed assembly information is shown in Supplemental Table 4. *^b^* http://rice.uga.edu/. *^c^* Homogous DNA sequences detected by NCBI megablast. *^d^* Approximate location in Nipponbare genome (IRGSP 1.0 pseudomolecule) (kb). The detailed assembly information is shown in Supplemental Table 4.
